# Molecular basis for potentiation of Cx36 gap junction channel conductance by *n*-alcohols and general anesthetics

**DOI:** 10.1042/BSR20171323

**Published:** 2018-02-08

**Authors:** Vytautas Raškevičius, Vaidas Jotautis, Lina Rimkutė, Alina Marandykina, Mintautė Kazokaitė, Visvaldas Kairys, Vytenis Arvydas Skeberdis

**Affiliations:** 1 Institute of Cardiology, Lithuanian University of Health Sciences, Kaunas LT-50162, Lithuania; 2 Institute of Biotechnology, Vilnius University, Vilnius LT-10257, Lithuania

**Keywords:** Connexin 36, disulphide bonds, gap junction, isoflurane, n-alcohols

## Abstract

In our recent study, we have demonstrated that short carbon chain *n*-alcohols (up to octanol) stimulated while long carbon chain *n*-alcohols inhibited the conductance of connexin (Cx) 36 (Cx36) gap junction (GJ) channels. In contrast, GJ channels composed of other types of Cxs all were inhibited by *n*-alcohols independent of their carbon chain length. To identify the putative structural domains of Cx36, responsible for the dual effect of *n*-alcohols, we performed structural modeling of Cx36 protein docking with hexanol and isoflurane that stimulated as well as nonanol and carbenoxolone that inhibited the conductance of Cx36 GJs and revealed their multiple common docking sites and a single pocket accessible only to hexanol and isoflurane. The pocket is located in the vicinity of three unique cysteine residues, namely C264 in the fourth, and C92 and C87 in the second transmembrane domain of the neighboring Cx36 subunits. To examine the hypothesis that disulphide bonding might be involved in the stimulatory effect of hexanol and isoflurane, we generated cysteine substitutions in Cx36 and demonstrated by a dual whole-cell patch-clamp technique that in HeLa (human cervix carcinoma cell line) and N2A (mouse neuroblastoma cell line) cells these mutations reversed the stimulatory effect of hexanol and isoflurane to inhibitory one, typical of other Cxs that lack respective cysteines and a specific docking pocket for these compounds. Our findings suggest that the stimulatory effect of hexanol and isoflurane on Cx36 GJ conductance could be achieved by re-shuffling of the inter-subunit disulphide bond between C264 and C92 to the intra-subunit one between C264 and C87.

## Introduction

**Figure 1:**
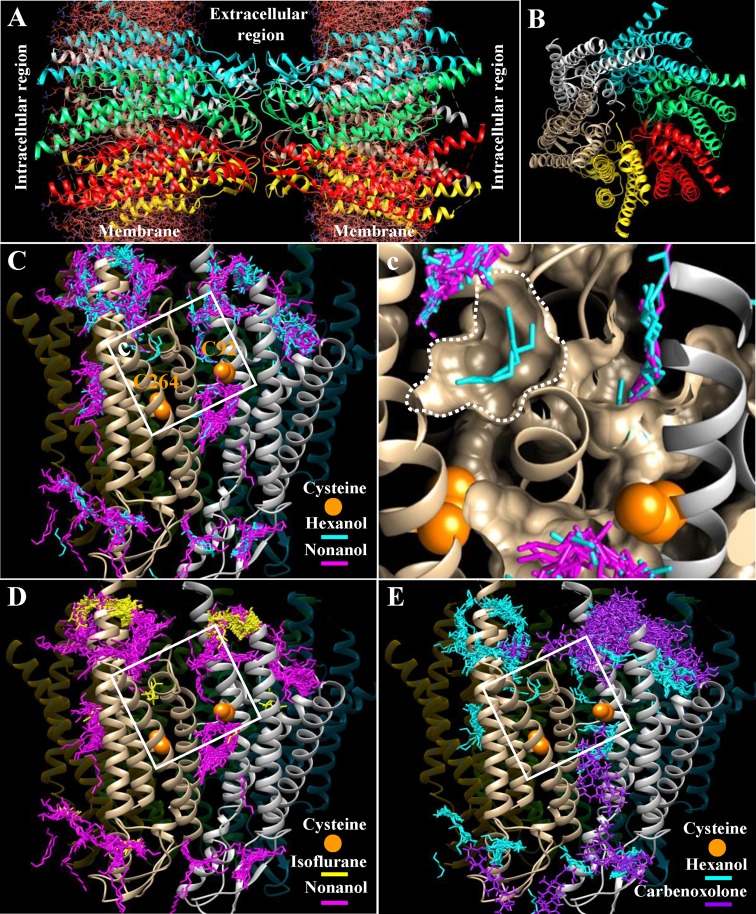
Cx36 contains multiple docking sites for both hexanol and nonanol and a unique docking pocket for hexanol (**A**) Front view of Cx36 GJ channel (without cytoplasmic loop and C-terminal segment) in ribbon representation following Dr Maeda model of Cx26 [26](RSCB Protein Data Bank ID: 2ZW3). Each Cx36 hemichannel is composed of six Cx36 subunits (indicated in different colors). (**B**) Side view of Cx36 GJ channel showing the arrangement of the transmembrane helices TM1 to TM4. (**C**) A unique docking pocket of hexanol in the TM4 of Cx36 is located close to C264 of TM4 and C92 of TM2 of the neighboring Cx36 subunits. (**c**) View of the hexanol docking pocket at higher magnification. (**D**) R-isoflurane docks at the same pocket as hexanol. (**E**) Carbenoxolone does not dock at the unique hexanol docking pocket.

Gap junction (GJ) channels (Figure 1A,B) are composed of two apposed hemichannels in the contiguous cells and provide a direct pathway for electrical and metabolic intercellular communication. Each hemichannel consists of six connexin (Cx) subunits. The family of Cx genes comprises 21 members in the human genome. Various tissues express different Cxs that form GJs with unique single channel properties and responses to transjunctional voltage (V_j_), intracellular Ca^2+^, pH_i_, phosphorylation, and chemical reagents (reviewed in references [7,35,37,45]). Recently we have demonstrated that Cx36 distinguishes from other Cxs by its opposite response to widely used GJ uncouplers, such as *n*-alcohols or general anesthetics. For instance, while these agents inhibit Cx45 GJs [40], they strongly potentiate Cx36 GJs [28]. Importantly, the effect of *n*-alcohols is carbon chain length dependent. Short carbon chain *n*-alcohols (up to octanol) stimulate, and long carbon chain *n*-alcohols inhibit the conductance of Cx36 GJs [28]. We hypothesized that stimulatory and inhibitory effects of *n*-alcohols may originate from their interaction with different domains of Cx36 protein as, for instance it has been shown in nicotinic acetylcholine receptor channels [49]. Structural modeling of Cx36 protein docking with hexanol that stimulates and nonanol that inhibits the conductance of Cx36 GJs revealed multiple common docking sites for hexanol and nonanol and a single pocket accessible only to hexanol. The pocket is located in the vicinity of two unique cysteine residues, C264 in the fourth and C92 in the second transmembrane domain (transmembrane domain (TM) TM 4 and TM2, respectively) of the neighboring Cx36 subunits. The presumptive proximity of C92 and C264 suggested that putative disulphide bonding between Cx36 subunits might modulate GJ conductance as it has been demonstrated, for instance in acid-sensing ion channels [50]. In addition, we identified C87 in TM2 as a possible candidate for disulphide bond re-shuffling. Cysteines in the extracellular loops that are involved in hemichannel docking and form intra-connexin disulphide bonds are not accessible to external reducing agents [10]. The same may apply to the cysteines residing in the TM domains of Cx36 because the pilot experiments in which we tested the effect of reducing agent DTT on Cx36 GJ conductance did not provide an unambiguous answer. Therefore, we generated single and double cysteine substitution(s) in Cx36 by replacing C87 and C264 with serine and C92 with valine to minimize the local volume changes. C92V mutation was chosen following the analysis of the Cx36 structure. C92 faces the membrane; therefore, it was reasonable to substitute it by valine, a hydrophobic amino acid, because serine could cause larger changes in local conformation. We demonstrate by dual whole-cell patch-clamp experiments that these mutations reversed the stimulatory effect of hexanol and isoflurane to inhibitory one, typical to other Cxs that lack respective cysteines or/and a specific docking pocket for these compounds.

## Materials and methods

### Cell lines and culture conditions

Experiments were performed on HeLa (human cervix carcinoma cell line, ATCC CCL-2) and N2A (mouse neuroblastoma cell line, ATCC CCL-131) cells transfected with Cx36 tagged with the GFP (Cx36-EGFP). The HeLa cell line and the pIRESpuro2_mCx36-EGFP vector were obtained from the laboratory of Dr F. Bukauskas (Albert Einstein College of Medicine, New York, U.S.A.). N2A (purchased from Sigma–Aldrich, Germany) or HeLa cells were transiently transfected with Cx36-EGFP or its mutants using Lipofectamine 2000 (Invitrogen, U.S.A.) and following the manufacturer’s transfection protocol. Cells were grown in DMEM containing 10% FBS, 100 U/ml penicillin, and 100 μg/ml streptomycin (Gibco Laboratories). Typically, the experiments were performed on the second day after cell passage.

### Construction of Cx36-EGFP mutants

In order to generate point mutants, we used a site-directed mutagenesis kit (QuickChange Lighting Site-Directed Mutagenesis Kit, Agilent Technologies, Santa Clara, CA, U.S.A.). The kit utilizes the three-step procedure protocol comprising the following: (i) PCR with mutagenic primers, (ii) digestion of template following on PCR with DpnI restriction endonuclease, and (iii) the transformation of XL10-Gold ultracompetent cells. Mutagenic primers containing desired mutation(s) are equal in length and anneal to the same sequence on opposite DNA strands. In Table 1, underlined nucleotides of primers indicate introduced codons of amino acids.

**Table 1:** Primers used for construction of Cx36-EGFP mutants

Number	Primer names	Forward and reverse primers	Length, bp
1	C87S-forward	5′-ccagatcataatggtgagcacccccagtctctg-3′	33
	C87S-reverse	5′-cagagactgggggtgctcaccattatgatctgg-3′	
2	C92V-forward	5′-ggtgtgcacccccagtctcgtttttatcacctattctgtg-3′	40
	C92V-reverse	5′-cacagaataggtgataaaaacgagactgggggtgcacacc-3′	
3	C264S-forward	5′-tttgctgtgagcggcatttctgtggtgctcaatc-3′	34
	C264S-reverse	5′-gattgagcaccacagaaatgccgctcacagcaaa-3′	

For the introduction of two point mutations in the same vector of DNA we performed double-stage PCR using templates from the previous mutagenesis, i.e. the single point mutant template was utilized for construction of double point mutant. Agarose gels were captured using the Herolab gel documentation system, Biometra TI 1, UV transilluminator, and BioDoc Analyze 2.0 software. Clones were tested using restriction endonucleases and afterward potentially positive clones were sequenced for the final confirmation of introduced mutation(s) (Sequencing Centre of the Institute of Biotechnology, Vilnius University, Lithuania).

### Structural modeling

Cxs (Cx30.2, Cx36, Cx43, Cx45, and Cx47) homology modeling was carried out with MODELLER (version 9.10) [47], using a Cx26 crystal structure [26] as a template. Computational modeling of molecular docking was performed using the Vdock program [20]. Proteins and small molecules were modeled using CHARMM22 (Chemistry at Harvard Macromolecular Mechanics, version 22) [25] and CHARMm with Momany–Rone charges [30], respectively. The ligand parameters were generated using Discovery Studio Visualizer (version 3.5, Accelrys Software Inc., San Diego, U.S.A.). Distance-dependent dielectric approximation was employed to model solvent effects [23]. Hexanol, nonanol, R-isoflurane, S-isoflurane, and carbenoxolone were selected as docking ligands. All molecular structures were created as the Brookhaven Protein Data Bank ‘pdb’ or MDL Molfile ‘mol’ molecular data format files and converted into Charmm ‘crd’ and molecular topology ‘top’ files using the Discovery Studio 3.5 Visualizer, UCSF Chimera (version 1.8.1, University of California, San Francisco, U.S.A.) [36] and VMD (Visual Molecular Dynamics, version 1.9.2) [17] programs and custom scripts for docking with Vdock graphical user interface. Most docking settings were left as default as provided in the Vdock program except 300 docking conformations were saved instead of usual 20, and the genetic algorithm for the conformational search was disabled. Such settings enabled more thorough and unbiased exploration of multiple binding pockets on the surface of the receptor, especially if the lowest energy minima were narrow in the conformational space [21]. The docking box was set up in such way that docking explored the entire surface of two neighboring Cx36 subunits. All modeling results were visualized with the UCSF Chimera system or PyMOL (Molecular Graphics System, version 1.8, Schrödinger, LLC, U.S.A.).

All molecular dynamics (MD) calculations were performed with the GROMACS (Groningen Machine for Chemical Simulations, version 2016.2) [2]. Time steps of 2-fs duration were used for all MD simulations. PLUMED (version 2.3.0) [44] was used for steered MD (SMD). We used the Amber99 SB-ILDN [24] force field for the protein and the SPCE [5] for all water molecules of the system. The electroneutrality of the system was achieved by adding Cl^−^ ions. Prior to MD simulations, the steepest descent minimization with up to 50000 steps was being carried out until maximum force declined below 1 MJ.mol^–1^.nm^–1^. Then, two 100-ps equilibrations (isothermal–isochoric and isothermal–isobaric) with constrained with protein heavy atoms and 1-ns SMD production run were carried out with a force constant between C92 and C264 sulphur atoms set to 1 Mcal.mol^–1^.nm^–2^ for an initial approach into contact range. During the production of SMD runs, all position restraints were released. An additional 100-ps SMD run with a force constant set to 1 Gcal.mol^−1.^nm^−2^ was used to overcome steric repulsion between the sulphur atoms and to model a covalent bond. The ligands were treated with the general AMBER force field (GAFF) [42] for organic molecules using the ACPYPE program (version 01/17/2017) that is based on the Antechamber software [41,46]. RMSD was calculated with GROMACS between starting point of SMD run and all other points after protein superposition.

### Electrophysiological measurements

For electrophysiological recordings, the cells grown on to glass coverslips were transferred to the experimental chamber with a constant flow through perfusion system mounted on the stage of the inverted microscope Olympus IX81 equipped with the Orca-R^2^ cooled digital camera, fluorescence excitation system MT10 (Olympus Life Science Europa Gmbh, Hamburg, Germany), and fluorescence imaging system XCELLENCE. Appropriate excitation and emission filters (Chroma Technology, Brattleboro, VT, U.S.A.) were used to image Cx36-EGFP GJs. Junctional conductance between cells was measured in selected cell pairs using the dual whole-cell patch-clamp technique (MultiClamp 700B with digitizer Digidata 1440A; Molecular Devices, Inc., U.S.A.). Cell-1 and cell-2 of a cell pair were voltage clamped independently at the same holding potential. By applying voltage ramp (ΔV_j_) from –10 to 10 mV in cell-1 and keeping the other constant, junctional current ΔI_j_ (GJ current) was measured as the change in current in cell-2. Thus, gap junction conductance (g_j_) was obtained from the ratio −ΔI_j_/ΔV_j_, and the negative sign indicates that junctional current measured in cell-2 is oppositely oriented to the one measured in cell-1. The experiments were performed at room temperature in a modified external Krebs–Ringer solution: 140 mM NaCl, 4 mM KCl, 2 mM CaCl_2_, 1 mM MgCl_2_, 5 mM glucose, 2 mM pyruvate, 5 mM HEPES (pH 7.4). Patch pipettes were pulled from borosilicate glass capillary tubes with filaments and filled with internal solution: 130 mM KCl, 10 mM Na aspartate, 2 mM MgATP, 1 mM MgCl_2_, 0.2 mM CaCl_2_, 2 mM EGTA, 5 mM HEPES (pH =7.3). To minimize the effect of series resistance on the measurements of g_j_, we maintained pipette resistances below 3 MOhms.

### Statistical analysis

Data are expressed as mean of at least four independent experiments ± S.E.M. The unpaired Student’s *t* test was used for quantitative evaluation. Differences were considered statistically significant at *P*<0.05.

## Results

### Docking sites of hexanol and nonanol on the Cx36 protein

To identify the putative structural domains of Cx36, responsible for a dual effect of *n*-alcohols, we performed molecular docking of hexanol that stimulated and nonanol that inhibited the conductance of Cx36 GJ channels. The docking revealed multiple common docking sites for hexanol and nonanol, and a single pocket accessible only to hexanol (Figure 1C,c).

**Figure 2:**
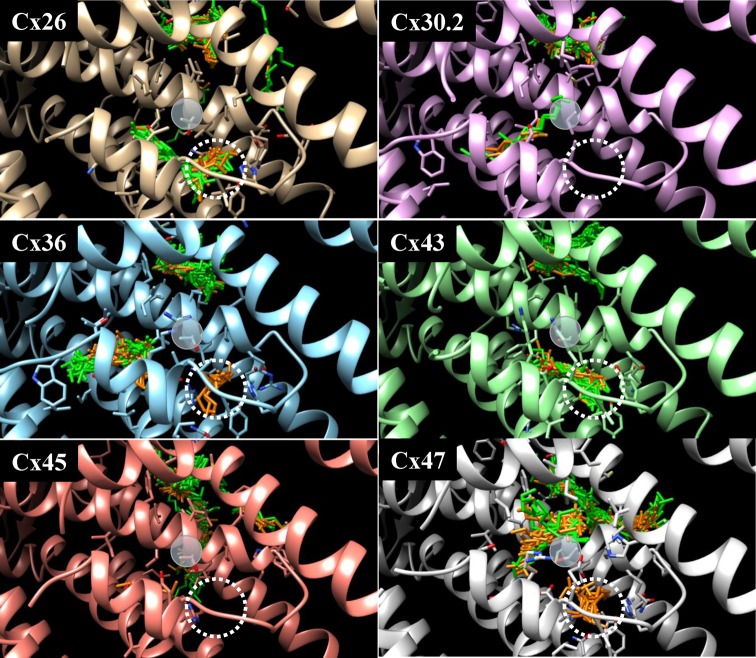
Docking of hexanol (orange) and nonanol (green) to different Cxs The docking pocket of hexanol on Cx36 or an analogous site on the other tested Cxs is indicated by a dotted circle. A transparent disc indicates a site of C264 or an analogous amino acid on the other tested Cxs.

We assessed the stability of hexanol binding to this pocket by MD simulations. As can be seen in Supplementary Figure S1 (additional file 1), stabilization of conformation was achieved during the first 5 ns of simulation, and later on the complex structure was stable and no dissociation occurred during the entire 100-ns simulation. Such validation also was used by other authors [43]. The pocket is located in the vicinity of two cysteine residues, C264 in the TM4 and C92 in the TM2, of the neighboring Cx36 subunits, respectively. Notably, hexanol molecules but not nonanols also docked into an analogous pocket of Cx47 (Figure 2). However, the amino acid sequence in TM2 and TM4 is unique to Cx36 compared with Cx47 and other tested Cxs because of the presence of cysteines that presumably could form the inter-subunit disulphide bonds. This could explain the observation that except for Cx36, all other tested Cxs isoforms (Cx26, Cx30.2, Cx43, Cx45, and Cx47) did not exhibit a dual, carbon chain-dependent response to *n*-alcohols [28].

Isoflurane is a racemic mixture of (R)- and (S)-optical isomers [12]; however, only R-isoflurane was able to dock in the same hexanol-specific pocket of Cx36 (Figure 1D).

Differences in the specific isoflurane docking pocket in the examined Cxs are shown in Supplementary Figure S2 (additional file 2). All Cxs were aligned, and isoflurane was placed inside the unique docking pocket in the same conformation as it docks into Cx36. Side chains in a close range with isoflurane are shown as sticks for every Cx for comparison. For docking of the specific compound, the dimensions of the pocket should be of an appropriate size. Larger dimensions make it nonspecific while lower dimensions create significant Van der Waals atom surface overlaps between amino acid residues and the ligand. Only Cx36 and Cx47 possess a specific docking pocket for isoflurane or hexanol (Figure 2). In both, the docking pocket is composed of nine amino acids (Supplementary Figure S2, additional file 2). The positions of Gln^212^, Tyr^91^, Glu^11^, Glu^12^, Lys^22^, His^16^, Tyr^209^, and Arg^216^ in Cx47, and Gln^202^, Phe^93^, Glu^12^, Ala^13^, Arg^24^, His^18^, Tyr^199^, and Arg^206^ in Cx36 are homologous. As determined by the docking procedure, access of hexanol and isoflurane to the analogous site is blocked by Arg^143^ and Tyr^136^ in Cx30.2, by Tyr^17^ and Lys^162^ in Cx43, and by Tyr^91^ and Arg^184^ in Cx45. In Cx26, the docking pocket is not sterically blocked as in these Cxs, but it lacks an aromatic ring as Phe^93^ inside Cx36 or Tyr^91^ inside Cx47, and therefore, it cannot accommodate hexanol or isoflurane.

**Figure 3:**
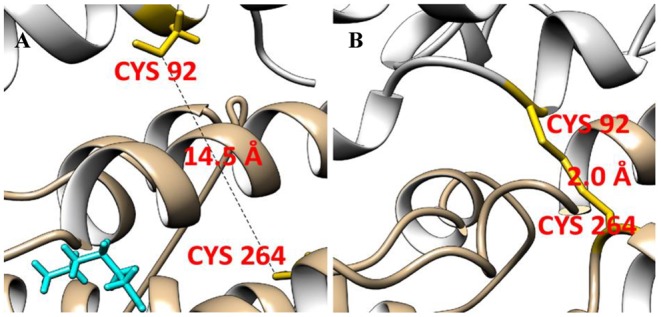
Putative disulphide bond formation between neighboring subunits of Cx36 Distance between C92 and C264 of the neighboring Cx36 subunits is 14.5 Å in the homology model (**A**) and approaches 2.0 Å in the SMD model (**B**). Hexanol is shown in cyan color.

The estimated disulphide bond length is ~2 Å [32]. It is important to stress that a distance between C264 and C92 of the neighboring Cx36 subunits provided by a homology model was >10 Å (Figure 3A); however, this model was developed using the structure of Cx26 [26] that differs from Cx36 by its amino acid sequence and electrophysiological properties. In addition, in contrast with Cx26 and all other Cx isoforms, Cx36 has two cysteines in TM2. We presumed that real distances between C264 and C92 might be much shorter. To examine a possibility that the molecular structure of Cx36 subunits in general can allow their conformation bringing cysteines together, we performed SMD. C264 and C92 approached each other into contact range (3.6 Å) during an initial SMD run, and as it is shown in Figure 3B, a distance between them could be reduced to 2.0 Å during the additional SMD run.

During SMD simulation, work increased from 0 to 334 kJ/mol within the first 100 ps and remained constant over the remaining simulation time. Supplementary Figure S3A (Additional file 3) shows that 334 kJ/mol of energy was sufficient to overcome structural hindrances precluding the disulphide bond formation. Such an energy value is comparable with single covalent bond strength.

Thus, these results suggested that formation of disulphide bonds between C264 and C92 was likely, and to further examine this possibility, we constructed C264S and C92V single and double mutants of Cx36-EGFP, and examined their properties in HeLa and N2A cells by the dual whole-cell patch-clamp technique.

### The effects of hexanol and nonanol on GJ conductance of Cx36-EGFP and its mutants

**Figure 4:**
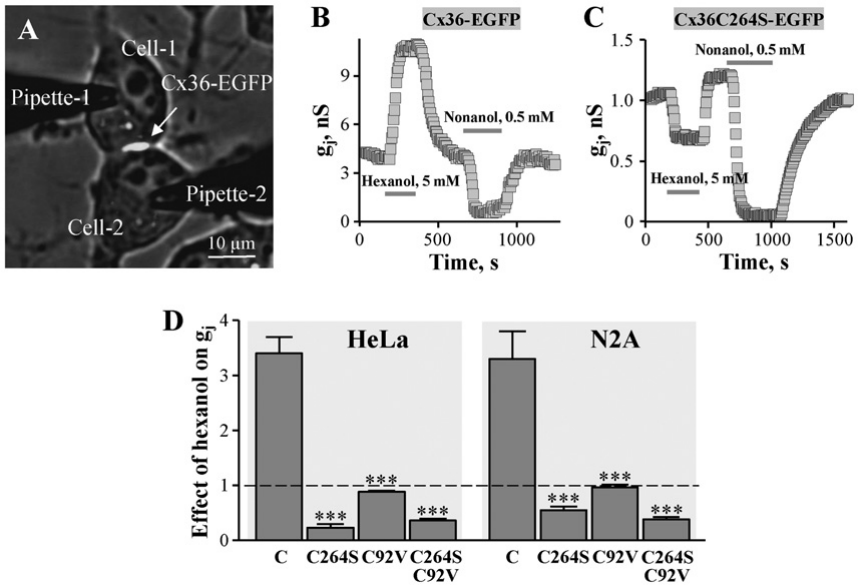
The effect of hexanol on GJ conductance of Cx36-EGFP and its mutants (**A**) Two abutted N2A cells expressing Cx36-EGFP GJ plaque. Junctional current was measured by applying voltage ramps from –10 to 10 mV to the cell-1 and measuring current response in the cell-2. Junctional conductance g_j_ was obtained from the ratio –ΔI_j_/ΔV_j_. (**B**) Typical effect of hexanol and nonanol on Cx36-EGFP GJ conductance in N2A cells – g_j_ is potentiated by hexanol and inhibited by nonanol. (**C**) Typical effect of hexanol and nonanol on Cx36C264S-EGFP GJ conductance in N2A cells – g_j_ is inhibited by both hexanol and nonanol. (**D**) Summary of the effects of hexanol on GJ conductance of Cx36-EGFP and its mutants in HeLa and N2A cells. The effect of hexanol relatively to initial g_j_ (indicated by dotted line) of Cx36-EGFP (indicated as C), Cx36C264S-EGFP, Cx36C92V-EGFP, and C36C264S/C92V-EGFP GJs was 3.4 ± 0.3 (*n*=9), 0.23 ± 0.06 (*n*=4), 0.88 ± 0.02 (*n*=4), and 0.36 ± 0.03 (*n*=8), respectively, in HeLa cells, and 3.3 ± 0.6 (*n*=6), 0.55 ± 0.06 (*n*=7), 0.96 ± 0.05 (*n*=6), and 0.38 ± 0.04 (*n*=8), respectively, in N2A cells. ***P<0.001, compared with Cx36-EGFP.

In our earlier experiments, we used HeLa cells exogenously expressing Cx36 or Cx36-EGFP [28]. HeLa cells express low levels of endogenous Cx45 that occasionally forms low transjunctional conductance GJs [9]. Therefore, in addition to HeLa cells we used other communication-deficient N2A cell line. Junctional conductance was measured between the selected Cx36-EGFP-expressing cell pairs (Figure 4A). Under control conditions, g_j_ varied from 1 to 15 nS depending on a GJ plaque size. A typical experiment (Figure 4B) demonstrates that while hexanol (5 mM) induced ~2.5-fold stimulation of Cx36-EGFP GJ conductance, it was almost completely blocked by nonanol (0.5 mM). The stimulatory effect of hexanol on Cx36 GJ conductance was not affected by insertion of EGFP in the C-terminus of Cx36 since hexanol stimulated to the similar extent the g_j_ of exogenous Cx36 GJ channels in HeLa cells (2.7 ± 0.4-fold; *n* 4) and endogenous Cx36 GJ channels in mouse pancreatic *β*-cells (2.2 ± 0.6-fold; *n* 7) [28] expressing solely Cx36 [31,39]. In contrast, the stimulatory effect of hexanol was absent from cells expressing Cx36C264S-EGFP (Figure 4C). In these cells both hexanol and nonanol inhibited g_j_ presumably through a common docking site(s) on Cx36. Similar results were obtained in both HeLa and N2A cells (summary is presented in Figure 4D) just in HeLa cells the inhibitory effect of hexanol was slightly stronger suggesting that endogenous Cx45 GJs may have manifested. These differences could be reduced using siRNA against endogenous Cx45 (data not shown). The stimulatory effect of hexanol was absent also from both types of cells expressing Cx36C92V-EGFP or double-mutant Cx36C92V/C264S-EGFP (Figure 4D).

### The effect of isoflurane on GJ conductance of Cx36-EGFP and its mutants

**Figure 5:**
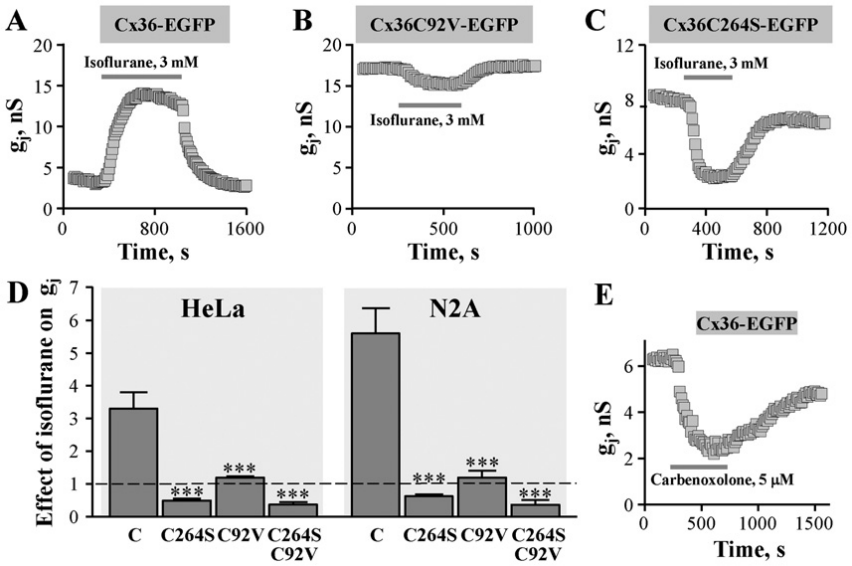
The effect of isoflurane on GJ conductance of Cx36-EGFP and its mutants Typical effects of isoflurane on Cx36-EGFP (**A**), Cx36C92V-EGFP (**B**), and Cx36C264S-EGFP (**C**) GJ conductance in N2A cells. (**D**) Summary of the effects of isoflurane on GJ conductance of Cx36-EGFP and its mutants in HeLa and N2A cells. The effect of isoflurane relatively to initial g_j_ (indicated by dotted line) of Cx36-EGFP (indicated as C), Cx36C264S-EGFP, Cx36C92V-EGFP, and C36C264S/C92V-EGFP GJs was 3.3 ± 0.5 (*n*=8), 0.49 ± 0.06 (*n*=9), 1.22 ± 0.04 (*n*=8), and 0.37 ± 0.08 (*n*=6), respectively, in HeLa cells, and 5.8 ± 0.8 (*n*=6), 0.62 ± 0.06 (*n*=4), 1.2 ± 0.2 (*n*=5), and 0.36 ± 0.14 (*n*=4), respectively, in N2A cells. ***P<0.001, compared with Cx36-EGFP. (**E**) Typical effect of carbenoxolone on GJ conductance of Cx36-EGFP in HeLa cells.

General anesthetics such as isoflurane and halothane uncouple cells expressing GJs at concentrations used for inhalation anesthesia [27]. However, isoflurane stimulated the Cx36-EGFP GJ conductance in HeLa and N2A cells (Figure 5A) suggesting that it may act through the same mechanism as hexanol. Structural modeling revealed that R-isoflurane (but not S-isoflurane) is capable of docking to Cx36 in the same unique pocket as hexanol and at multiple other docking sites as nonanol (Figure 1C). This suggested that stimulatory effect of isoflurane on Cx36 GJ conductance also could be exerted by counteracting the inter-subunit disulphide bond formation between C92 and C264. Indeed, the stimulatory effect of isoflurane was absent from cells expressing single mutants Cx36C92V-EGFP or Cx36C264S-EGFP, or double mutant Cx36C92V/C264S-EGFP (see Figure 5B–D).

Importantly, another nonselective inhibitor of GJ conductance carbenoxolone, a derivative of glycyrrhetinic acid, that did not fit to the hexanol docking pocket (Figure 1E), also did not stimulate but, like nonanol, reversibly inhibited g_j_ of Cx36-EGFP GJ channels (Figure 5E). G_j_ was inhibited to 40 ± 4% (*n*=7) of control with 5 µM and was completely blocked with 100 µM of carbenoxolone (not shown).

### The effect of *n*-alcohols and isoflurane on GJ conductance of Cx31-EGFP

**Figure 6:**
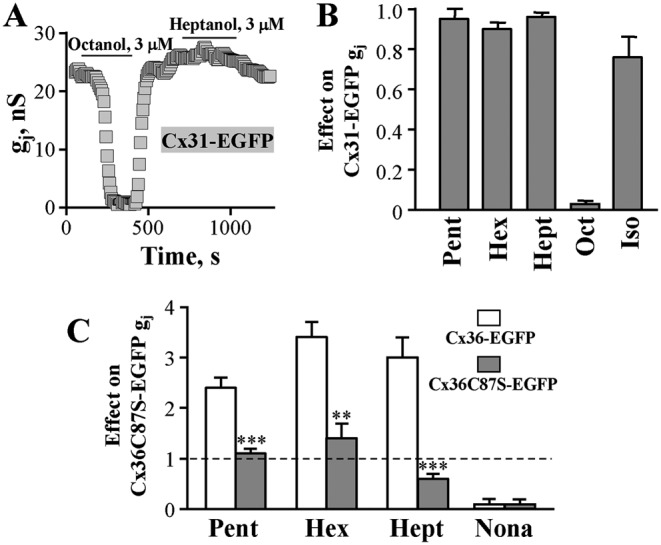
The effect of *n*-alcohols and isoflurane on GJ conductance of Cx31-EGFP (**A**) Typical effects of octanol and heptanol on Cx31-EGFP GJ conductance in HeLa cells. (**B**) Summary of the effects of *n*-alcohols and isoflurane on GJ conductance of Cx31-EGFP in HeLa cells. The effect of pentanol (5 mM), hexanol (5 mM), heptanol (3 mM), octanol (3 mM), and isoflurane (3 mM) was 0.95 ± 0.50 (*n*=5), 0.90 ± 0.03 (*n*=8), 0.96 ± 0.02 (*n*=4), 0.03 ± 0.01 (*n*=6), and 0.75 ± 0.10 (*n*=4), respectively. (**C**) Comparison of the effects of pentanol (5 mM), hexanol (5 mM), heptanol (3 mM), and nonanol (0.5 mM) on g_j_ of Cx36-EGFP and Cx36C87S-EGFP GJ channels. The effect was 2.4 ± 0.2 (*n*=7) and 1.1 ± 0.1 (*n*=9); 3.4 ± 0.3 (*n*=9), and 1.4 ± 0.3 (*n*=10); 3.0 ± 0.4 (*n*=7) and 0.6 ± 0.1 (*n*=8); 0.1 ± 0.1 (*n*=10), and 0.1 ± 0.1 (*n*=5), respectively. **P<0.005; ***P<0.001.

Some other Cxs, such as Cx30.3, Cx31, and Cx31.1, possess cysteines in TM2 and TM4 at similar positions as Cx36. Therefore, we chose human Cx31 to compare its responses to *n*-alcohols and isoflurane with those of Cx36-EGFP. As determined by homology modeling, an estimated distance between C86 and C198 of Cx31 is 11.48 Å, and it can be reduced by SMD; however, the access of *n*-alcohols and isoflurane to the analogous site on Cx31 is occluded by Tyr^16^ (Supplementary Figure S2, Additional file 2). Consequently, short C-chain *n*-alcohols and isoflurane had no stimulatory effect on its g_j_ (Figure 6A). Surprisingly, they also had no inhibitory effect while longer C-chain *n*-alcohols completely blocked Cx31 GJ conduction (Figure 6B). This suggests that the accessibility of *n*-alcohols to the inhibitory site(s) on Cx31-EGFP may depend on their C-chain length; however, confirmation of this hypothesis requires detailed examination and is within scope of our future investigations.

### C87 as a candidate for disulphide bond re-shuffling

Recent evidences suggest that disulphide bonds in proteins are highly dynamic (reviewed in [34]). Disulphide bonds may react with thiols within proteins even in the absence of enzymes and re-shuffle spontaneously or on an external stimulus, inducing changes in protein conformation and possibly function [3,22]. To examine this possibility, we performed homology modeling search for possible candidate cysteines. Cx36 has one more cysteine in its TM2 domain, and quite unexpectedly to us, we found that it was located even closer to C264 of the same subunit (11.2 Ǻ) than C92 of the neighboring subunit (Supplementary Figure S3B, additional file 3). C264 and C87 approached each other into a contact range (3.6 Ǻ) during an initial SMD run (Supplementary Figure S3A), as in the case of C264 and C92, and a distance between them could be reduced to 2.0 Ǻ during the additional SMD run. Moreover, the stimulatory effect of pentanol, hexanol, and heptanol was abolished in the Cx36C87S-EGFP mutant while the inhibitory effect of nonanol remained unchanged (Figure 6C).

These observations support our hypothesis that the potentiation of Cx36-EGFP GJ conductance by *n*-alcohols and anesthetics involves the specific binding site and re-shuffling of the inter-subunit disulphide bond between C264 and C92 to the intra-subunit one between C264 and C87. Other possibilities of re-shuffling (intra-subunit bonding of C87 and C92 or inter-subunit bonding of C264 and C87) are hardly possible due to unfavorable orientations of cysteine residues.

## Discussion

Neurological actions of alcohols and anesthetics are linked with pentameric ligand-gated ion channels (pLGICs) such as γ-aminobutyric acid receptor type A (GABA(A)), glycine, and nicotinic acetylcholine receptors [8,11,13,51]. Alcohols and anesthetics evoke their effects by binding to the specific residues in the transmembrane domain that lines the ion channel pore [13,38], and it is proposed that pLGICs contain intrasubunit-inhibitory and inter-subunit-potentiating sites [16,33]. GJs or, in other words, electrical synapses in the CNS can be inhibited by general anesthetics suggesting that they may play a role in anesthesia [19]. Cx36 that is a dominant neuronal GJ protein also has been shown to be implicated in mechanisms of general anesthetic action [18,48]. For instance, isoflurane or propofol was more potent in Cx36 knockout animals [18], and the authors suggested that Cx36 GJs may act as buffer against effects of anesthetic drugs. This observation may harmonize with our recent discovery [28] that in contrast with other Cxs, the activity of Cx36 GJ channels is not inhibited but can be several times potentiated by *n*-alcohols up to octanol and, as we demonstrate in the present study, by isoflurane. This property is particularly interesting because *n*-alcohols with a longer carbon chain such as nonanol inhibited GJs composed of Cx36 suggesting that like pLGICs it may have inhibitory site(s) accessible to all *n*-alcohols and a potentiating site accessible only to short carbon chain alcohols. In the present study, we demonstrate by structural modeling that such putative stimulatory site may exist in the TM4 of Cx36 (Figure 1). Moreover, we noticed that C264 of the TM4 is located in the proximity to C92 of the TM2 of the neighboring Cx36 subunit suggesting that disulphide bonds may form between them. As it has been shown with other ion channels [6,15], such disulphide bonding resulted in decreased channel activity and could be abolished by reducing agents. Therefore, we hypothesized that *n*-alcohols and anesthetics may potentiate Cx36 GJ channels by initiating disulphide bond re-arrangement. Indeed, mutation(s) of C92 or C264 completely abolished the stimulatory effect of both hexanol, a representative of short carbon chain alcohols, and isoflurane, and did not change the effect of nonanol, a representative of long carbon chain alcohols. As a possible mechanism, we proposed that potentiation may be achieved due to re-shuffling of the inter-subunit disulphide bond between C264 and C92 to the intra-subunit one between C264 and C87. We supported this presumption by demonstrating that even though Cx31 with single cysteine in its TM2 (C86) and TM4 (C198) (see below) might form putative disulphide bonds, it has no options for disulphide re-shuffling as a response to external stimulus and consequently is not potentiated by *n*-alcohols and isoflurane.

It is worth noting that predicted distances (>10 Å) between C264 and C92 of the neighboring Cx subunits exceed the estimated length of S–S bonds (~2 Å). However, as a template for structural modeling of Cx36, we used the only determined so far structure of Cx26 [26]. TM4 and TM2 of Cx36 differs in the amino acid sequence from those of Cx26 and contains two cysteines in TM2 while Cx26 has no cysteines in its TM2 [4]:

TM2 of mCx26: LWALQLIMVSTPALLVAM
TM2 of mCx36: WVFQIIMVCTPSLCFITYSV
TM2 of hCx31: LWALQLIFVTCPSLLVIL
TM4 of mCx26: VFTVFMISVSGICILLNIT
TM4 of mCx36: VFLVFMFAVSGICVVLNLA
TM4 of hCx31: IFTYFMVGASAVCIVLTIC

For that reason, we made a presumption that in reality Cys^264^ and Cys^92^ may stand closer and demonstrated that by SMD applying the reasonable energy. Alternatively, hexanol and isoflurane may act through another mechanism involving cysteines. For instance, alcohols and anesthetics may potentiate glycine or GABA(A) receptors by binding at the conserved inter-subunit cavity [38] or they, covalently binding to cysteine residues introduced into TM2 of glycine and GABA(A) receptor subunits, irreversibly enhance a receptor function [29]. In addition, it is possible that a unique disulphide bond has to exist for the potentiation of Cx36 GJ conductance by *n*-alcohols and anesthetics.

General anesthetics have minimal effects on bilayer properties at clinically relevant concentrations indicating that their effects rather involve direct protein interactions, but higher concentrations of certain anesthetic agents can alter lipid bilayer properties [14]. However, short carbon chain alcohols (up to hexanol) cause an increase in free (interstitial) volume of phospholipid membranes, whereas longer alcohols penetrating into the membrane core cause a decrease in it [1]. Consequently, membranous proteins can be either stimulated or inhibited what is in agreement with our results. Our hypothesis is supported by additional observations: (i) unique docking pocket of hexanol is absent from other types of tested Cxs (Cx26, Cx30.2, Cx43, Cx45) that did not exhibit a dual, carbon chain-dependent response to alcohols [28]; (ii) even though Cx47 possesses a similar hexanol-docking pocket, it has no cysteines in its TM2 (as well as all other tested Cxs) that could form intra- or inter-subunit disulphide bonds, and consequently is not stimulated by hexanol or isoflurane [28]; (iii) Cx31 with single cysteine in its TM2 and TM4 does not possess a docking pocket for hexanol and isoflurane, and consequently is not stimulated by these compounds; (iv) carbenoxolone, another nonselective inhibitor of GJ conductance, that did not fit to the hexanol docking pocket of Cx36-EGFP also did not stimulate but, like nonanol, reversibly inhibited g_j_ of Cx36-EGFP GJ channels.

The specific amino acid distribution may at least in part explain the preferences of the specific docking site for hexanol and isoflurane (Supplementary Figure S2, additional file 2); however, further investigations are necessary to relate the docking sites of used compounds to their effects on the Cx36 GJ channel function. First, determination of the Cx36 structure is necessary instead of using the Cx26 homology model. Second, MD would help determine the putative docking sites of compounds, evoking the observed phenomenon, more precisely. Finally, mutagenesis of these sites and patch-clamp experiments will plausibly link the structural domains with responses of Cx36 GJs to *n*-alcohols and anesthetics.

Our findings propose a novel mechanism by which *n*-alcohols and general anesthetics may potentiate Cx36 GJ conductance by binding to the specific site, located at the TM4 domain and inducing disulphide bond re-arrangement (putative model is shown in Supplementary Figures S3C,D, additional file 3).

### Supporting information

## Abbreviations

Cx: connexin
GABA(A): γ-aminobutyric acid receptor type A
GJ: gap junction
g_j_: GJ conductance
GROMACS: Groningen Machine for Chemical Simulations
HeLa: human cervix carcinoma cell line
I_j_: GJ current
MD: molecular dynamics
N2A: mouse neuroblastoma cell line
pLGIC: pentameric ligand-gated ion channel
SMD: steered MD
TM: transmembrane domain
V_j_: transjunctional voltage
