# Supplementary figures and images for: Molecular basis for potentiation of Cx36 gap junction channel conductance by n-alcohols and general anesthetics
Source: Biosci Rep. 2018 Feb 8;38(1):BSR20171323. doi: 10.1042/BSR20171323 (PMC5803492; doi:10.1042/BSR20171323)

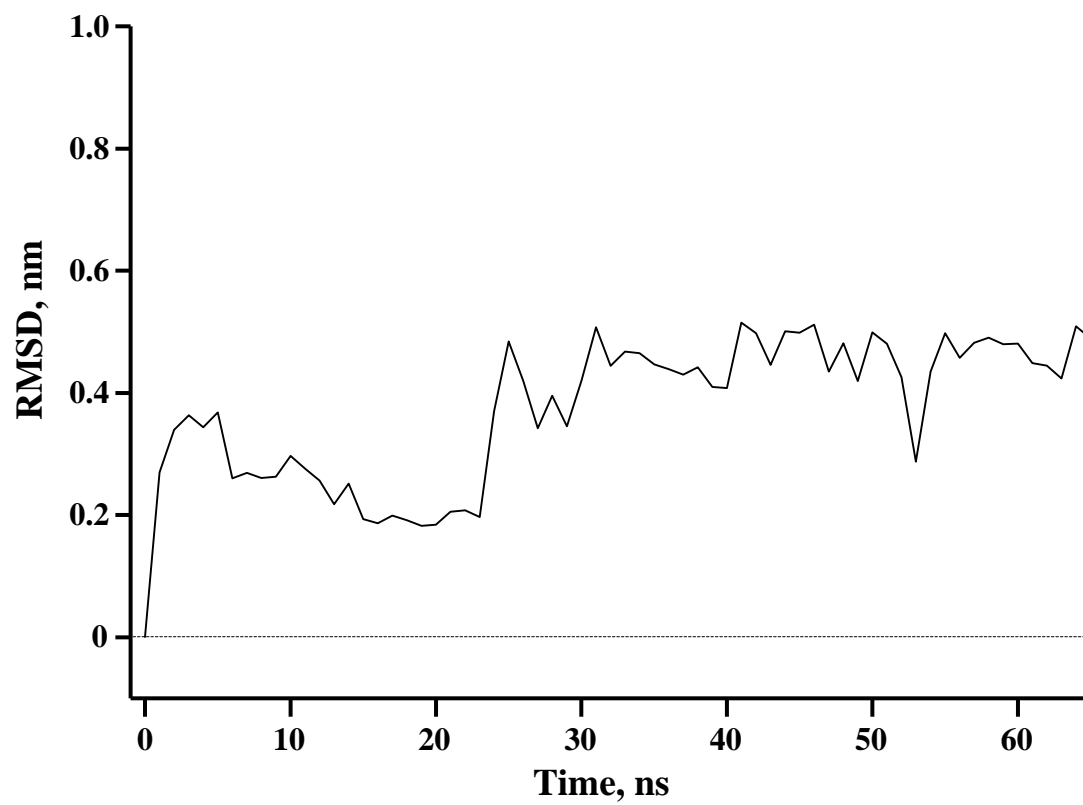

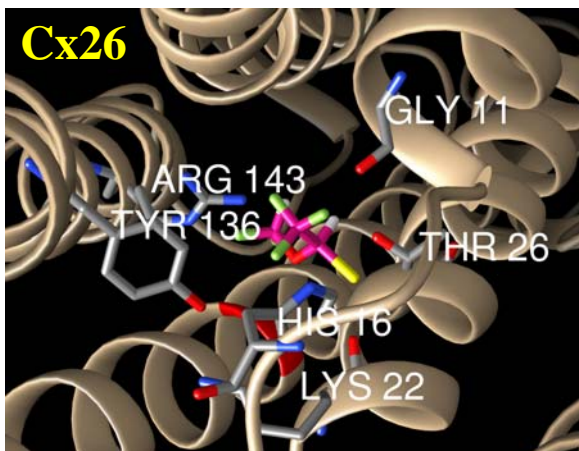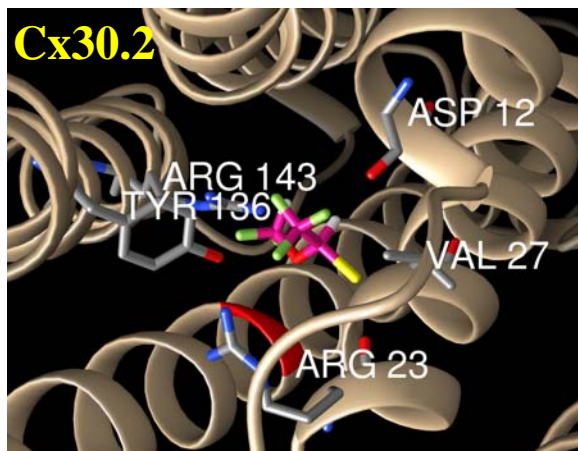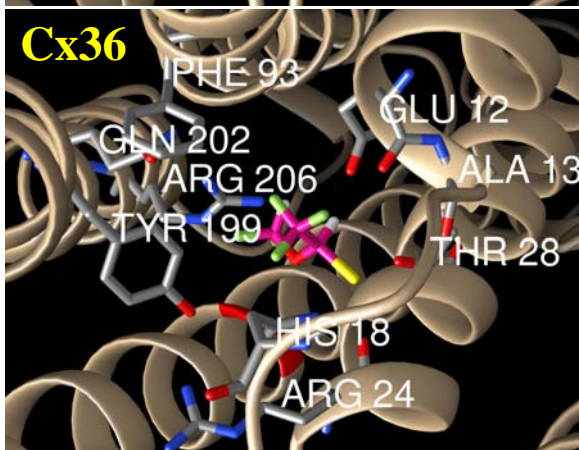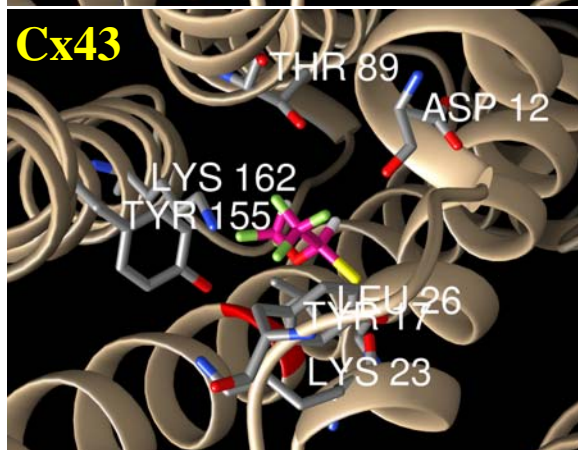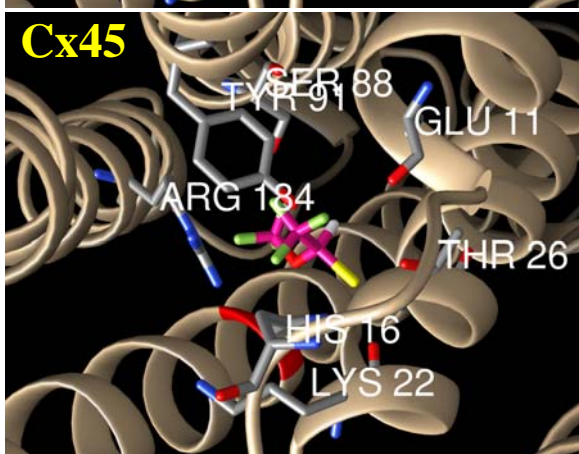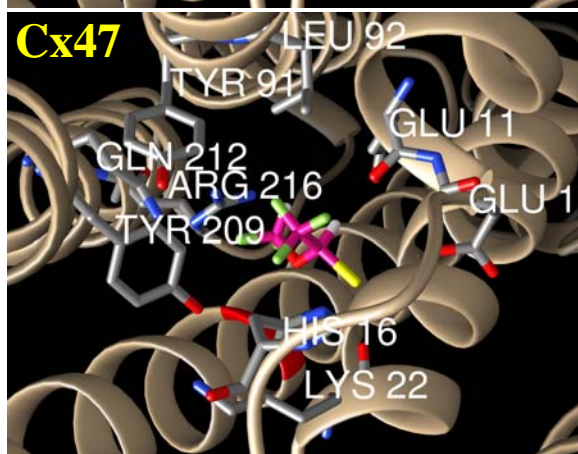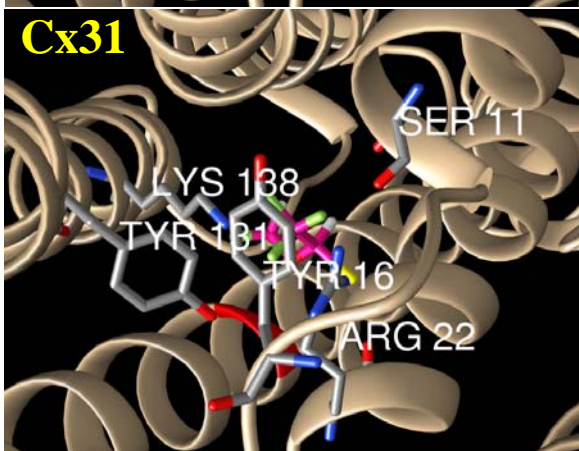

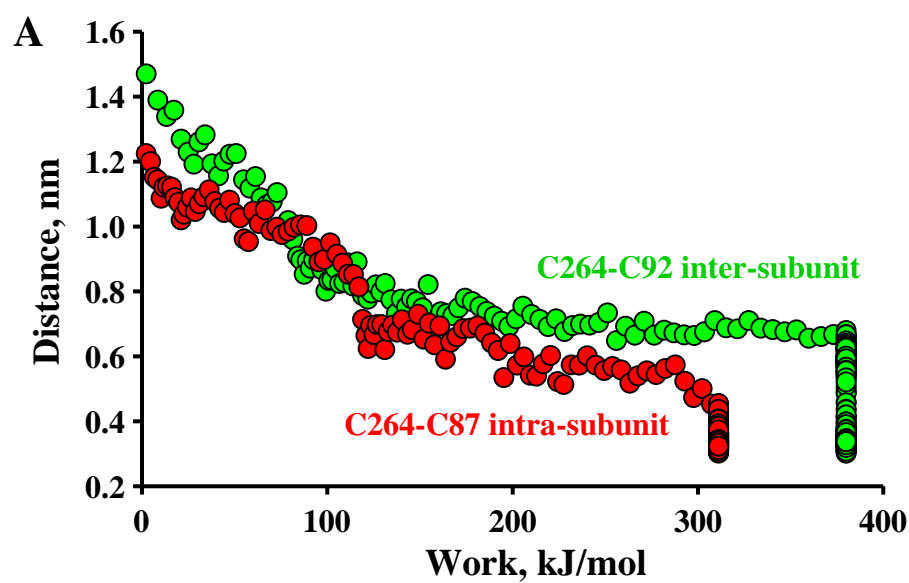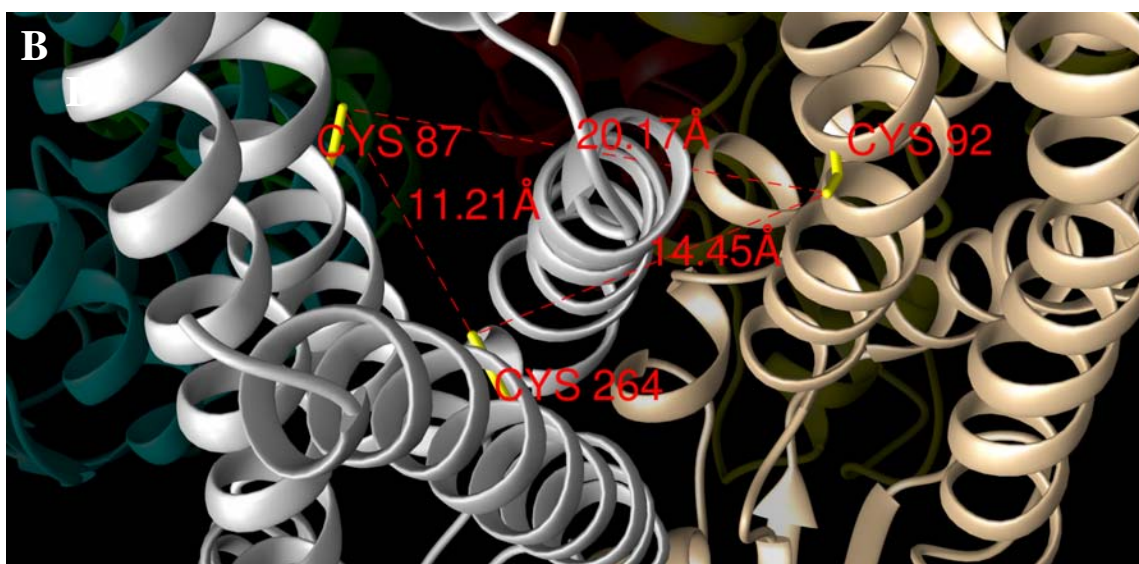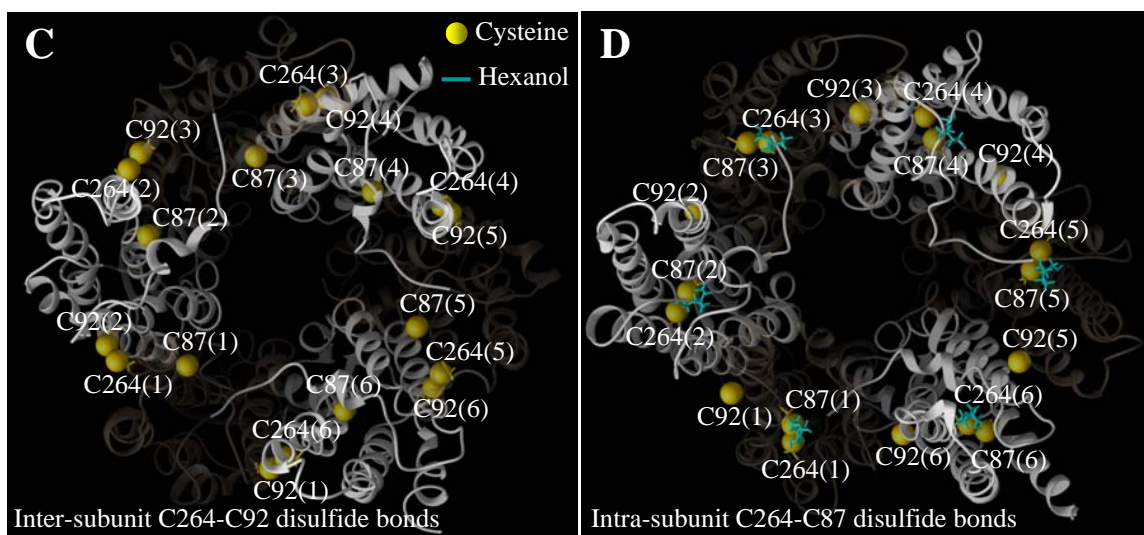

Supplement: Supplementary file 1 [file bsr20171323_Supp1.pdf]
